# Macadamia Nut-Induced Anaphylactic Shock Requiring Repeated Intramuscular Adrenaline Administration in a Three-Year-Old Girl

**DOI:** 10.7759/cureus.60858

**Published:** 2024-05-22

**Authors:** Takashi Kiyama, Katsuhiko Kitazawa

**Affiliations:** 1 Department of Pediatrics, Asahi General Hospital, Asahi, JPN

**Keywords:** food allergy, prick test, adrenaline, macadamia nut, anaphylactic shock

## Abstract

Cases of macadamia nut-induced anaphylactic shock have been rarely reported. We report the case of a three-year-old girl with anaphylactic shock who presented with generalized erythema two hours after ingesting macadamia nuts. She required two doses of intramuscular adrenaline for the treatment of anaphylactic shock. The diagnosis of macadamia nut allergy was confirmed by a prick-by-prick skin test using roasted and raw macadamia nut paste extracts and elevated serum macadamia nut-specific immunoglobulin E (IgE) levels. Appropriately using a prick-by-prick test may contribute to accurately diagnosing macadamia nut allergy, thus preventing the unnecessary avoidance of other nuts. Considering the potential for severe shock induced by macadamia nut allergy, vigilant monitoring of blood pressure changes is imperative in children presenting with immediate-type allergic reactions, such as vomiting and skin symptoms, following macadamia nut ingestion.

## Introduction

In the United States, 1-2% of the population is estimated to be allergic to peanuts, tree nuts, or both [1]. Although macadamia nuts are widely used in confectionery products, macadamia nut allergy is rare, accounting for <5% of tree nut allergic patients [1]. Few case reports have documented the incidence of anaphylaxis caused by consumption. Therefore, clinical information regarding severe macadamia nut allergy is limited. The low diagnostic accuracy of specific immunoglobulin E (IgE) testing for macadamia nut allergy poses a significant challenge in diagnosing this condition [2]. Herein, we report the case of a child with anaphylactic shock who presented with generalized erythema following the consumption of macadamia nut-containing confectionery. The patient was subsequently diagnosed with macadamia nut allergy by prick-by-prick testing using macadamia nut paste.

## Case presentation

The patient was a three-year-old girl with no history of an allergic disease. She had previously consumed peanuts and almonds but had not been exposed to macadamia nuts. On the presentation day, the child consumed ice cream infused with macadamia nuts for the first time. Ten minutes after ingestion, she exhibited irritability and vomiting and eventually took a nap. Upon awakening two hours after ingestion, the parents observed that the child exhibited generalized erythema and excessive drooling. Consequently, they promptly brought the patient to the hospital’s emergency department. During examination, the patient presented with a temporal temperature of 36.7 °C, a pulse rate of 167 beats per minute, a systolic blood pressure of 60 mm Hg, and oxygen saturation of 92% while breathing ambient air. She exhibited spontaneous eye opening but did not move in response to verbal stimulation. Physical examination revealed generalized erythema and marked bilateral conjunctival hyperemia. She experienced vomiting following food consumption, and food poisoning caused by infectious agents was considered a differential diagnosis. However, because the patient also had non-gastrointestinal symptoms such as erythema and marked conjunctival injection, food poisoning was highly unlikely. Based on these clinical findings, including hypotensive shock and mucocutaneous manifestations, the patient was diagnosed with anaphylactic shock, which was suspected to be triggered by allergens present in the ice cream. An intramuscular dose of adrenaline (0.01 mg/kg) was administered alongside oxygen supplementation (10 L/min by face mask) and rapid infusion of 20 mL/kg normal saline. Ten minutes after the initial adrenaline administration, the patient demonstrated a heart rate of 130/min and systolic blood pressure of 80 mmHg. Because the patient continued to exhibit generalized erythema, a second intramuscular adrenaline dose was administered. Subsequently, the patient had a heart rate of 120/min, systolic blood pressure of 90 mmHg, and erythema resolution. Following admission, an investigation by the ice cream manufacturer revealed that macadamia nuts were the only ingredient of concern. The patient recovered and was discharged the following day.

**Figure 1:**
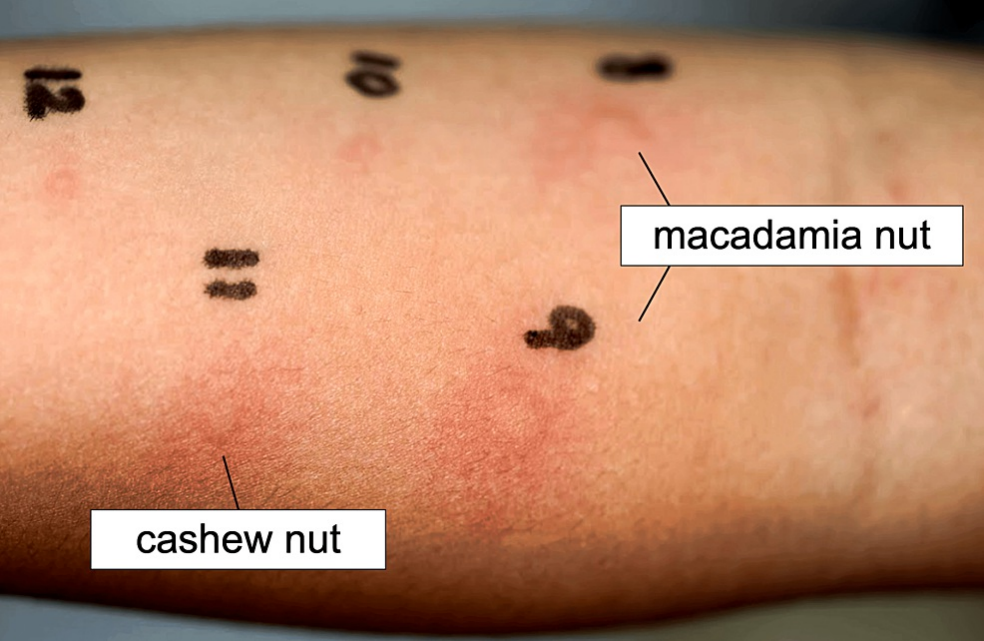
Photo depicting the reaction on the patient’s forearm after the prick test No. 8 (Roasted macadamia nut paste), No. 9 (Raw macadamia nut paste), and No. 11 (Roasted cashew nut paste) had diameters of 5.0mm, 5.0mm, and 3.5mm, respectively.

After discharge, the cause of anaphylaxis was investigated for further confirmation. The results of the specific IgE tests are shown in Table 1. We measured allergen-specific IgE levels in nuts previously associated with cross-reactivity with macadamia nuts [3-6]. The allergen-specific IgE levels were 0.66 UA/mL in hazelnuts, 2.20 UA/mL in cashew nuts (Ana o 3), and <0.10 UA/mL in other nuts. Subsequently, 1.26 UA/mL of macadamia nut-specific IgE conjugate (ImmunoCAP ®︎ f345) was submitted to Phadia Assay Support as a research item. A skin prick test was performed. Extracts were prepared from roasted macadamia nuts, raw macadamia nuts, hazelnuts, roasted cashew nuts, and roasted peanuts, and each paste was used for the prick test. The nut extracts were prepared according to the report of a previous study [7]. The nuts were crushed in a mortar and pestle and centrifuged thrice at 12,000 rpm for 15 minutes at 4°C. The extract was then filtered through an Advantec DISMIC-25 filter. In a previous study, filters with a pore diameter of 0.45 μm were used. Because this specific filter was unavailable in the hospital, another filter with a pore diameter of 0.25 μm was used. Her parents were informed of the risk of anaphylaxis before the prick test, which was performed using readily available emergency medications. The results of the prick tests are shown in Table 2 and Figure 1. A positive reaction was defined as a wheal diameter greater than 3 mm or more than half of the wheal diameter of the positive control (score 2+). The patient tested positive for roasted and raw macadamia nuts, with a swelling size of 5 mm after 15 min (4+). Exposure to roasted cashew paste elicited a 3+ reaction, with a swelling size of 3.5 mm. Exposure to other products (roasted hazelnut and peanut paste) resulted in the same swelling size as that of the negative control, indicating a negative result. Based on these findings, the patient was diagnosed with macadamia nut allergy. The patient was also considered to be allergic to cashew nuts. Hence, macadamia and cashew nuts were removed from the diet, whereas other nuts previously consumed without adverse reactions were considered safe and continuously consumed. The patient was advised to consume hazelnuts and peanuts at home during the day if eaten for the first time. An adrenaline auto-injector was prescribed as emergency medication. Since then, the patient has remained free from any immediate allergic episodes to nuts other than macadamia and cashew nuts.

**Table 1:** Specific IgE test results Macadamia nuts-specific immunoglobulin E conjugate (ImmunoCAP®︎ f345) was submitted to Phadia Assay Support as a research item. Classes are determined as follows: Class 0: <0.10 kU/L - Negative, Class 0/1: 0.10-0.34 kU/L - Borderline/Equivocal, Class 1: 0.35-0.69 kU/L - Equivocal, Class 2: 0.70-3.49 kU/L - Positive, Class 3: 3.50-17.49 kU/L - Positive, Class 4: 17.5-49.9 kU/L - Strongly positive, Class 5: 50.0-100 kU/L - Strongly positive, Class 6: >100 kU/L - Strongly positive. IgE: Immunoglobulin E

Specific IgE	IgE class	UA/mL
Alder	0	< 0.10
Birch	0	< 0.10
Hazelnut	1	0.66
Brazil nut	0	< 0.10
Almond	0	< 0.10
Coconut	0	< 0.10
Cashew nut (Ana o 3)	2	2.20
Walnut (Jug r 1)	0	< 0.10
Macadamia nut	2	1.26

**Table 2:** Results of the skin prick test The skin prick test was performed using a bifurcated needle. A positive reaction was defined as a wheal diameter greater than 3 mm or more than half of the wheal diameter of the positive control (score 2+). Scores are determined as follows: equal to the negative control: -, less than one-half, more significant than the negative control: 1+, one-half of the positive control: 2+, wheals equal to the positive control: 3+, twice the wheals of the positive control: 4+.

No	Materials	Diameter (mm)	Score
	Nut extracts	2.0	-
8	Roasted macadamia nut paste	5.0	+4
9	Raw macadamia nut paste	5.0	+4
10	Roasted hazelnut paste	2.0	-
11	Roasted cashew nut paste	3.5	+3
12	Roasted peanut paste	2.0	-

## Discussion

A three-year-old girl experienced anaphylactic shock following the ingestion of macadamia nuts and required emergency management, including repeated intramuscular injections of adrenaline. Anaphylaxis is an acute, potentially life-threatening systemic allergic reaction with a wide range of clinical manifestations [8]. In children, although anaphylaxis is often caused by the ingestion of food allergens, including tree nuts, few cases of allergy to macadamia nuts have been reported [6,7,9-11]. It is widely acknowledged that monitoring allergic symptoms is essential when introducing peanuts to children [12]; however, there is a lack of awareness regarding macadamia nuts. Additionally, allergic reactions are challenging to recognize when vomiting is the first symptom. Parents may need to be educated on recognizing food allergy-induced symptoms in children, even those with no history of allergic diseases.

Our literature search from 1991 to 2013 yielded 25 individuals with documented cases of macadamia nut allergy [6,7,9-11]. Of the 25 patients, 20 (80%) presented with skin symptoms, 16 (64%) with oral mucous membrane symptoms, 16 (62%) with respiratory symptoms, 5 (20%) with gastrointestinal symptoms, and 7 (28%) with shock symptoms. Diagnosing anaphylaxis in children with no history of allergic disease remains challenging, and vomiting following initial food ingestion could serve as an early indicator of anaphylaxis. In this case, the patient exhibited generalized erythema and was diagnosed with anaphylactic shock two hours after ingestion of a macadamia nut. It remains unclear whether these manifestations stemmed from a biphasic reaction, as the patient fell asleep immediately after vomiting, and other symptoms, including skin rash, were not observed by her parents.

One reason for the under-reporting of macadamia nut allergy is the challenging nature of diagnosing this condition. Several diagnostic methods have been investigated, including specific IgE testing, most of which utilized the ImmunoCAP®︎ f345 conjugate. However, the measurement of IgE specific to macadamia nuts does not always predict clinical allergy and may lead to false-negative results [2]. The cutoff value for predicting anaphylaxis by macadamia nut-specific IgE testing was >3.76 UA/mL [13]. However, the current case report reported a cut-off value of 1.26 UA/mL, indicating that the macadamia nut-specific IgE value may not always predict anaphylaxis in certain patients. A recent study identified Maci 1 and Maci 2 as macadamia nut allergens, suggesting a more accurate diagnosis of macadamia nut allergy using Maci 1- and Maci 2-specific IgEs may be achieved [14]. Although specific IgE testing may be insufficient for diagnosing macadamia nut allergy, prick tests may provide reliable diagnostic information. Macadamia nut extract is often used as a diagnostic allergen because of the lack of commercially available allergen extracts for diagnosing macadamia nut allergies [7]. A positive prick-by-prick test using macadamia nut paste confirmed the diagnosis of macadamia nut allergy. However, the complexity of preparing nut extracts limits their broad applicability in clinical practice for patients suspected of having macadamia nut allergy. Although macadamia extracts are preferred in allergy testing, they are challenging to produce. Furthermore, macadamia nut allergy testing using the extract method suggested the possibility of certain false negatives. The prick-by-prick test using the macadamia nut paste, on the other hand, is easier and more accurate. Children allergic to certain nuts are frequently advised to eliminate all nuts [15]. The prick-by-prick test can be instrumental in preventing the unnecessary elimination of nuts by patients with macadamia nut allergies.

## Conclusions

We treated a three-year-old girl with macadamia nut-induced severe anaphylactic shock that required repetitive adrenaline administration. The prick-by-prick test using macadamia nut extract was useful for accurately diagnosing macadamia nut allergy and preventing the unnecessary removal of other nuts. In emergency departments, because macadamia nut-induced anaphylaxis can result in severe shock, blood pressure changes should be closely monitored in children presenting with immediate-type allergic reactions, such as vomiting and skin symptoms, following macadamia nut ingestion. Convenient and minimally invasive diagnostic tools, such as precise IgE determination and standardized skin prick testing, will be of great value in managing children suspected of having macadamia nut allergy.
